# Hepatocellular carcinoma with situs inversus totalis treated by caudate lobectomy: A case report

**DOI:** 10.1016/j.ijscr.2022.107204

**Published:** 2022-05-17

**Authors:** Amane Kitasato, Takayuki Miyoshi, Tatsuya Okamoto, Akira Yoneda, Hiroaki Takeshita, Tamotsu Kuroki

**Affiliations:** Department of Surgery, National Hospital Organization Nagasaki Medical Center, Ohmura, Japan

**Keywords:** Situs inversus, Hepatocellular carcinoma, Hepatectomy, SIT, HCC, Caudate lobectomy

## Abstract

**Introduction:**

Situs inversus totalis (SIT) is a congenital anatomical variant in which organs and vasculature are positioned in a mirror-image relationship to the normal condition. Therefore, the surgical procedures need to be carefully planned with these factors in mind.

**Case presentation:**

A 57-year-old man with SIT was diagnosed with a hepatocellular carcinoma (HCC) and was planned for caudate lobectomy. As preoperative preparation, 3D reconstructed images were created based on the contrast-enhanced CT images, and careful simulations were performed on the vascular anomalies and location of the tumor. There was a replaced left hepatic artery forming a common trunk with a left gastric artery. In addition, using media player software, a previous caudate lobectomy video was played in right and left inverted mode to simulate the abdominal surgical field image in SIT. The operative time was 285 min, and the blood loss was 440 ml. The preoperative careful simulation allowed us to proceed with the surgery without significant discomfort.

**Conclusion:**

Even in the case of hepatocellular carcinoma with SIT, hepatectomy for hepatocellular carcinoma can be safely performed by careful preoperative simulations.

## Highlights

• This is a case of caudate lobectomy for hepatocellular carcinoma with situs inversus totalis.
• We repeatedly watched a previous caudate lobectomy video played in right and left inverted mode prior to the surgery.
• With careful preoperative simulations, liver resection can be safely performed in patients with SIT.

## 1 Introduction

Situs inversus totalis (SIT) is a congenital anatomical variant, occurring in approximately 1 in 10,000 births, in which organs and vasculature are positioned in a mirror-image relationship to the normal condition. In addition, it is reported that cardiovascular, hepatobiliary, and splenic malformations with anomalies of abdominal vessels are increased in patients with SIT [1]. Therefore, the surgical procedures need to be carefully planned with these factors in mind. Here, we report a case of caudate lobectomy for hepatocellular carcinoma with SIT, including preoperative measures. This case report was drafted and submitted according to the SCARE guidelines [2].

## 2 Case presentation

**Fig. 1:**
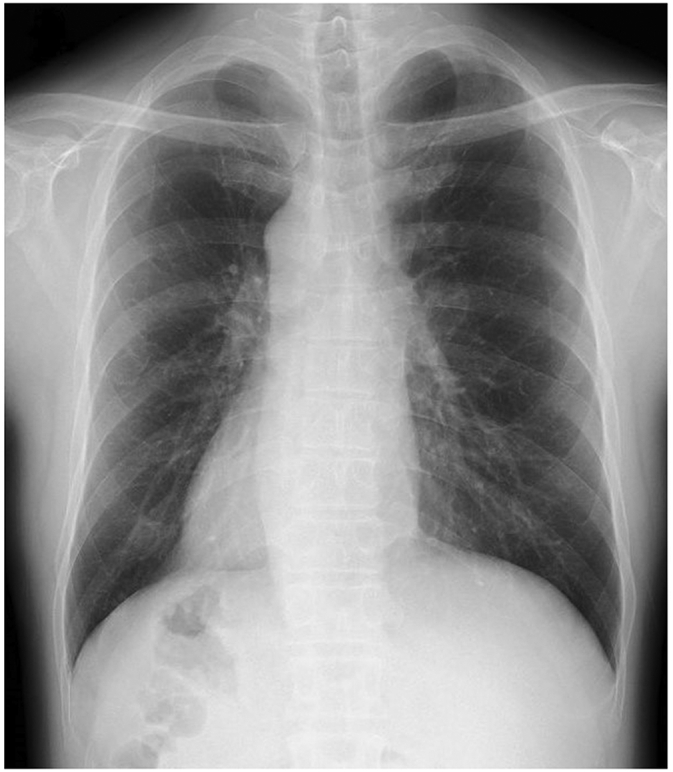
A chest X-ray showed dextrocardia.

**Fig. 2:**
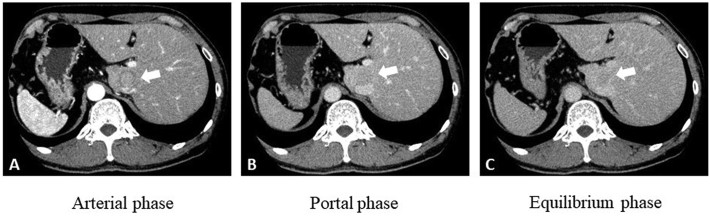
Preoperative computed tomography (CT). A: Arterial phase. B: Portal phase. C: Equilibrium phase. Enhanced CT showed a 2.5 cm nodule at the caudate lobe with early enhancement, followed by washout during portal and equilibrium phase (arrow). The tumor compressed the inferior vena cava dorsally.

A 57-year-old man with SIT, who was under follow up for chronic hepatitis B at our hospital, was diagnosed with a liver tumor during a routine examination. A chest X-ray showed dextrocardia (Fig. 1). A computed tomography (CT) scan showed a right-to-left reverse transposition of the organs in the thoracic cavity and the abdomen, and an abdominal contrast-enhanced CT scan revealed a 2.5 cm nodule at the caudate lobe with early enhancement, followed by washout during portal and equilibrium phase. The tumor compressed the hepatic portion of the inferior vena cava dorsally (Fig. 2). Tumor markers demonstrated an elevated protein induced by Vitamin K absence or antagonists-II (PIVKA-II) at 120 mAU/ml. Child-Pugh classification was A class and an uptake ratio of the liver to the liver plus heart at 15 min (LHL15) in technetium-99m-galactosyl human serum albumin (99mTc-GSA)scintigraphy was 0.919. The patient was diagnosed with a hepatocellular carcinoma (HCC) and caudate lobectomy was planned based on the tumor location and preserved liver function.

**Fig. 3:**
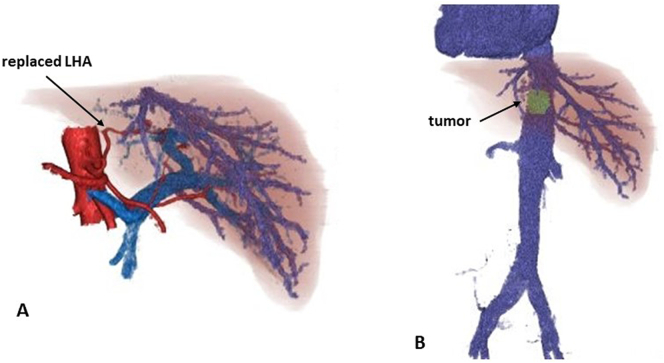
3D reconstructed images. A: A replaced left hepatic artery forming a common trunk with a left gastric artery was revealed. B: The tumor was located in the caudate lobe of the ventral aspect of the inferior vena cava.

**Fig. 4:**
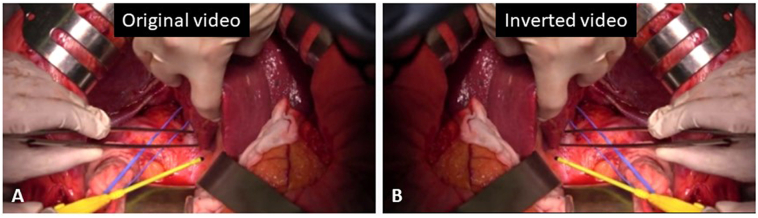
A previous caudate lobectomy video was played in right and left inverted mode using media player software. A: Original video. B: Inverted video.

Preoperative simulations: 3D reconstructed images were created based on the contrast-enhanced CT images, and careful simulations were performed on the vascular anomalies and location of the tumor (Zaio Workstation, Zaiosoft, Inc., Tokyo, Japan). There was a replaced left hepatic artery (LHA) forming a common trunk with a left gastric artery. No other major malformations were observed (Fig. 3). In addition, using media player software (VideoProc Converte™, Chengdu Digiarty Software, Inc., Chengdu, China), a previous caudate lobectomy video was played in right and left inverted mode to simulate the abdominal surgical field image in SIT (Fig. 4).

**Fig. 5:**
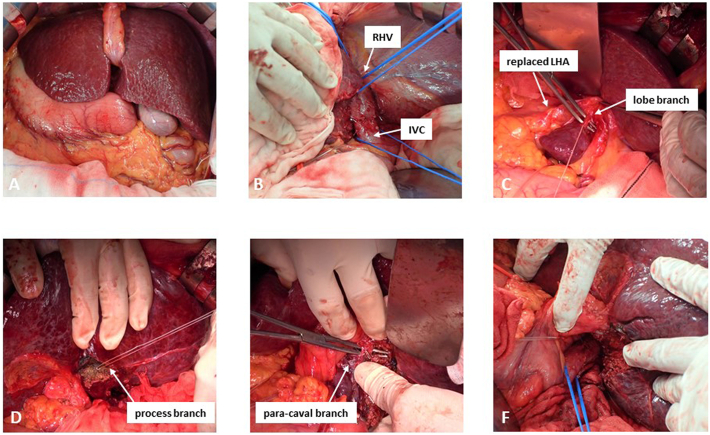
Intraoperative findings. A: Abdominal organs were positioned in the mirror-image to the normal position. B: Mobilization of the right lobe, and taping of the right hepatic vein and inferior vena cava. C: Ligation and dissection of the lobe branches, and D: process branches, and E: para-caval branches. F: After completion of caudate lobectomy.

During the operation, the operator first stood the right side of the patient for laparotomy and cholecystectomy, and then moved to the left side of the patient to mobilize the liver and thereafter continued the subsequent procedures. The right lobe was mobilized from retroperitoneum, and the inferior vena cava (IVC) was mobilized by ligating and dissecting the short hepatic veins. The right hepatic vein (RHV) and IVC were taped, respectively. Next, the replaced LHA was taped, and the trunk of the left and middle hepatic veins was taped after dissecting the lateral segment and dividing the ligamentum venosum (Arantius' duct). Due to the presence of the replaced LHA, separation of the Spiegel lobe from the IVC was performed from the right lobe side. After completely separation of the liver from the IVC except for the major hepatic veins, the lobe branches from the umbilical portion to the Spiegel lobe were divided. On the right lobe side, liver transection was conducted from the border of the posterior segment and the caudate process toward the sulcus of the ligament venosum, ligating and dividing the process branches and the para-caval branches, and the caudate lobectomy was completed (Fig. 5). The operative time was 285 min, and the blood loss was 440 ml. The preoperative careful simulation allowed us to proceed with the surgery without significant discomfort.

## 3 Discussion

In 1983, Kanematsu et al. reported the first case of liver resection for HCC patient with SIT [3], and we have been able to retrieve 18 reports of hepatectomy for SIT patients (Table 1). Of the 19 cases including the present case, 13 cases were HCC, 1 was combined hepatocellular and cholangio-cellular carcinoma, 3 were liver metastases, 1 was cavernous hemangioma of the liver, and 1 was a mass with unknown details. Liver resection is performed according to the lesion status, and several laparoscopic surgeries have recently been reported. To our knowledge, this is the first report of isolated caudate lobectomy in a patient with SIT. Including the present case, 6 of 14 reports in which anatomy of hepatic artery were described showed variations in hepatic artery [3], [4], [5], [6], [7], [8], [9], [10], [11], [12], [13], [14], [15]. These factors suggest that in patients with SIT, not only the mirror-image relationship of the organs to the normal condition, but also the anatomical variations of the vascular system, including the hepatic arteries, increase the difficulty of surgical procedures. Thus, preoperative detection of such vascular variations is important to perform the operation safely. In our case, we were able to confirm the replaced LHA forming a common trunk with a left gastric artery on 3D reconstructive images preoperatively, which allowed us to calmly respond to the patient during the surgery.

**Table 1:** Summary of 19 cases hepatectomy for SIT patients.

No.	Year	Author	Age/sex	Liver status	Disease	Location	Tumor Size	Surgical procedure	Variation in hepatic artery
1	1983	Kanematsu [3]	37/M	HBsAg+	HCC	Bil. lobe-multiple	–	Lt. lobectomy	None
2	1989	Kim [4]	66/F	HBsAg+	HCC	Rt. lobe	14 ∗ 12 cm	Rt. lobectomy	RHA arising from SMA
3	1996	Kamiike [5]	69/F	LC	Combined	S7	2.5 cm	Resection of postero-superior segment	None
4	1998	Iwakura [6]	63/M	LC-C	HCC	S6	1.5 cm	Lower posterior segmentectomy	None
5	1998	Hamada [7]	60/F	LC-C	HCC	S6-7	2.5 cm	Partial resection	None
6	1999	Seshimo [8]	70/M	LC-C	HCC	Lat. seg.	2 cm	Partial resection	CHA arising from SMA
7	2003	Goi [9]	72/F	Normal	LM	Post. upper segment	4.5 ∗ 5.0 cm	Resection of posterior upper segment	None
8	2004	Kakinuma [10]	70/F	LC-C	HCC	S8	3 cm	Partial resection	None
9	2006	Sawada [11]	76/M	Normal	HCC	Rt. lobe	Huge	Rt. tri-segmentectomy	None
10	2007	Matsukawa [12]	55/F	Normal	Cavernous hemangioma	Post. seg.	13 ∗ 12 cm	Extended posterior segmentectomy	CHA arising from SMA
11	2009	Uemura [13]	64/M	Normal	LM	Rt. lobe-multiple	–	Rt. lobectomy	PHA arising from SMA
12	2012	Harada [14]	59/M	Normal	HCC	Post. seg.	5 cm	Extended posterior segmentectomy	CHA arising from SMA LHA arising from LGA
13	2013	Uchiyama [16]	66/F	LC-C	HCC	Hilar lesion	5 cm	Enucleation	N/A
14	2013	Patel [15]	49/F	NASH	HCC	S7/8	12 ∗ 11 ∗ 9 cm	Rt. lobectomy	None
15	2017	Hong [18]	70/M	Alcoholic	Tumor	S5/6	5 cm	Lap rt. lobectomy	N/A
16	2017	Giuliani [17]	60/M	Normal	LM	S7	3 ∗ 2.4 cm	Lap partial resection	N/A
17	2018	Kimura [19]	75/M	nBnC	HCC	S4/8	2.7 cm	Lap partial resection	N/A
18	2020	Fu [20]	68/M	HBsAg+	HCC	S8, S6	3.6 cm	Rt. lobectomy	N/A
19	2022	Present case	57/M	HBsAg+	HCC	S1	2.5 cm	Caudate lobectomy	Replaced LHA

M; male, F; female, LC; liver cirrhosis, NASH; non-alcoholic steatohepatitis, HCC; hepatocellular carcinoma, Combined; combined hepatocellular and cholangiocellular carcinoma, LM; Liver metastases, Bil; bilateral, Rt; right, S; segment, Lat; lateral, Post; posterior, Lap; laparoscopic, RHA; right hepatic artery, SMA; superior mesenteric artery, CHA; common hepatic artery, PHA; proper hepatic artery, LHA; left hepatic artery, LGA; left gastric artery, N/A; not available.

In addition to a detailed understanding of anatomy and vascular variations, how preoperative simulations are performed is also important for smooth surgical procedure. Uchiyama et al. [16] described that creating the mirror image diagrams of the hepatic anatomy of their patients and making the operative plan preoperatively were helpful in safely performing a complex hepatectomy in a patient with SIT. Others, such as Giuliani et al. [17], reported that an intraoperative ultrasonography with the mode of scanning was switched from normal to reverse modality enabled to have on the screen the usual appearance of the intrahepatic anatomy, and was useful to reach a good resection line during totally laparoscopic liver resection. In the present case, we repeatedly watched a previous caudate lobectomy video played in right and left inverted mode prior to the surgery, which allowed us to perform the surgery without confusion in the actual mirrored surgical field.

## 4 Conclusion

Even in the case of hepatocellular carcinoma with SIT, hepatectomy for hepatocellular carcinoma can be safely performed by careful preoperative simulations.

## Consent

Written informed consent was obtained from the patient for publication of this case report and accompanying images. A copy of the written consent is available for review by the Editor-in-Chief of this journal on request.

## Ethical approval

No ethical approval was sought for this case report.

## Funding

This research did not receive any specific grant from funding agencies in the public, commercial, or not-for-profit sectors.

## Author contribution

AK and TK: study concept.

AK: data collection, writing the paper.

TM, TO, AY, HT, TK: reading and correcting the paper.

All authors: approving the final manuscript.

## Guarantor

Amane Kitasato.

## Registration of research studies

Not applicable.

## Declaration of competing interest

The authors declare that they have no competing interests.
